# The study of feasibility and acceptability of using HIV self-tests in high-risk Iranian populations (FSWs, MSM, and TGs): a cross-sectional study

**DOI:** 10.1186/s12954-022-00641-5

**Published:** 2022-06-03

**Authors:** Ghobad Moradi, Elnaz Ezzati Amini, Azam Valipour, Katayoon Tayeri, Parvin Afsar Kazerooni, Leila Molaeipour, Yousef Moradi

**Affiliations:** 1 Social Determinant of the Health Research Center, Research Institute for Health Development, Kurdistan University of Medical Sciences, Sanandaj, Iran; 2 HIV/AIDS Control Office, Center for Communicable Diseases, Ministry of Health, Tehran, Iran; 3 Department of Epidemiology, School of Public Health, Iran University of Medical Sciences, Tehran, Iran; 4 Department of Epidemiology and Biostatistics, School of Medicine, Kurdistan University of Medical Sciences, Sanandaj, Iran

**Keywords:** HIV self-test, Female sex worker, Transgender, Men who have sex with men, High-risk population, Iranian

## Abstract

**Background:**

This study aimed to evaluate the feasibility of using the HIV self-test in high-risk Iranian groups (MSM, FSWs, and TGs).

**Methods:**

This study was a mixed study designed as a quantitative–qualitative study conducted between October 1, 2020, and June 22, 2021, in Tehran and Karaj, Iran. The sample size needed for this study generally was 1000 people, including FSWs, MSM, and transgender individuals. Convenience and snowball sampling methods were used to collect the samples.

**Results:**

A total of 930 eligible respondents were enrolled in the study, of whom 456 (49%) were female and 49 (5.3%) were transgender (98% of TGs were male to female), and their mean age was 33.63 years (10.54 SD). The feasibility of using HIV self-tests in Iranian high-risk groups was significantly high. The majority of participants (97%) did not have any confidentiality problems while preparing or performing the test. In general, feasibility was assessed based on five questions. The overall feasibility score was 6.33 (0.824 SD). Taking tests, reading HIV test results, finding a safe place to do the test, and accessing HIV self-tests showed a high average.

**Conclusion:**

HIV self-testing was highly acceptable and feasible among high-risk populations, so routine HIV testing was efficiently possible.

## Introduction

According to the latest forecast of Spectrum, the HIV-positive population in Iran in 2019 was 59,314 people (range 32,685–125,636), including 862 children under 15 years old, 42,952 men over 15 years old, and 15,501 women over 15 years old with an estimated 4089 (range 36,354,507) new infections occurring during the same period. HIV prevalence in the general population in Iran remained low but stood at 4.32% among PWIDs in 2019, while before 2019, this rate was 13% [1, 2]. In general, HIV-related statistics have declined due to nationwide measures and interventions. However, a significant challenge with the HIV infection remains, which is the diagnosis of HIV in people who have not yet been referred to service centers for diagnostic procedures and receiving care services. Therefore, to achieve the goals set for HIV by 2030, a suitable solution must be developed to identify these people [3, 4]. Improving the diagnosis helps achieve national/global goals of HIV epidemic control [5]. The Islamic Republic of Iran, in all four UN special meetings on HIV in 2003, 2008, 2011, and 2016, has committed itself to HIV control and signed the declarations of these meetings. At the 2016 summit, the UN member countries and Iran pledged, based on the previous announcements, to accelerate the fight against AIDS through shortcuts to end the epidemic by 2030. These fast-track routes were divided into ten sections, the first of which was identifying patients and ensuring the access of HIV-infected people to treatment based on 90, 90, 90 strategies by 2020 [6–10]. Therefore, implementing a fast and safe way without stigma and discrimination to identify cases in high-risk groups can help end the HIV epidemic in the ten quick ways [11, 12]. Different diagnostic technologies in rapid diagnostic methods can help improve the diagnosis and ultimately control the disease. In March 2004, the HIV Antibody Rapid Detection Kit, introduced by the US Food and Drug Administration for salivary fluid samples in all individuals, became the first HIV self-test, or home testing [13–16].

In the following years, the quality of this test gradually improved, and its diagnostic value was also significantly enhanced. In Iran, with the control of the epidemic in injecting drug users (IDUs) and prisoners with the method of harm reduction, the transmission has led to high-risk groups. Of course, this also happened in many countries around the world. Given that most people at risk or high-risk groups, such as men who have sex with men (MSM), female sex workers (FSWs), and transgender people (TGs), may have not even once been diagnosed with HIV, their access to these tests is of great importance [12, 17, 18]. Many of these high-risk groups may be infected with HIV, but they are unaware of their status, so diagnosing the disease in these people can prevent the transmission of the infection to others. One of the reasons for not performing diagnostic tests by high-risk people may be the lack of easy access to these tests [19]. Therefore, by increasing the availability of the HIV self-test (HIVST) for high-risk groups, effective action can be taken in the early diagnosis of the disease in high-risk individuals to prevent its transmission to others [20, 21]. Even this program of HIV infection diagnosis can reduce the social stigma and fear in people while visiting centers and create more confidence in these groups to do the test. In pursuit of these goals, more high-risk groups will receive HIV/AIDS care and treatment [22–24]. If this program is accepted, the HIV testing for individuals and their sexual partners who may have never been tested for HIV will be provided and increased. Thirty-five countries worldwide have adopted the HIVST policy, and only 8–10 have implemented it [25, 26]. Iran is one of the countries, which has accepted the HIVST policy of the World Health Organization (WHO), but it has not yet taken any specific action to implement this test.

For this reason, this study was designed by the Ministry of Health and Medical Education of Iran to develop the necessary measures to implement the test in high-risk groups based on its results. Iran seeks to upgrade a codified action plan for feasibility and operationalizing HIVST in the general population and high-risk groups. This study aimed to evaluate the feasibility of using the HIV Self-Test in high-risk Iranian groups (MSM, FSWs, and TG people). The results of this study are expected to provide valuable evidence for the proper implementation of this program in the country.

## Materials and methods

This study was a mixed study designed as a quantitative–qualitative study conducted between October 1, 2020, and June 22, 2021, in Tehran and Karaj, Iran. This study has received the national ethical code of IR.MUK.REC.1399.133, and its population included participants of high-risk populations such as MSM, FSWs, and TGs, who signed an informed consent form before the study began.

Initially, its quantitative part was performed. In this study, no intervention was conducted, and the test was routinely distributed in the centers. After its distribution, the feasibility was measured in the groups of MSM, FSWs, and TGs. The sample size in this study was estimated at 385 people in each group by considering the minimum feasibility of 50% HIVST in high-risk groups (based on studies in other countries) [27–30], and the 95% confidence level (1.96). For the groups of FSWs and MSM, due to their predominance among the high-risk groups of Iran, 400 people were considered as the sample size for each group, and for the group of TGs, 200 people were considered because of the difficult access to this high-risk community in Iran. Finally, 1000 people were selected to participate in the study. Convenience and snowball sampling methods were used to collect the samples.

After selecting the sample size, the centers were selected for the study, including nine centers (four centers for MSM and TGs and five centers for FSWs) in the two provinces of Tehran and Alborz. Then, a one-day workshop was held to train experts in the selected centers. In this workshop, the study's aims, how to distribute the test, and how to educate high-risk people about the test and the report of the test result to the expert were trained. In addition to the workshop, a training pamphlet on how to test was distributed to the key groups during the test provision.

The inclusion criteria included lack of knowledge of high-risk people from their HIV/AIDS status, age over 18 years, satisfaction to participate in the study, men who had at least one sex with other men during the last 12 months (vulnerable men (MSM) and their sexual partners), women who had at least one sex in the past 12 months in exchange for money (vulnerable women (FSWs)), men who had at least one sex with vulnerable women (FSWs) during the last 12 months (sexual partners of vulnerable women (FSWs)), people diagnosed transgender by communicators (transgender people), and those who had at least one sex with transgender individuals over the past 12 months (transgender people’ sexual partners).

To achieve the research objectives, tools and methods of collecting data were used in different stages of the study. The first part was “the questionnaire of demographic characteristics,” the second part was “the questionnaire of knowledge and awareness,” the third part was “the questionnaire of acceptability, feasibility, and satisfaction,” and the fourth part was “the questionnaire of semi-structured interviews on identifying the benefits and disadvantages of the test and determining challenges in using this test in service provision centers.” In the quantitative part, the first three questionnaires were used. In order to better complete the information of high-risk groups and due to the COVID-19 pandemic, the questionnaires were designed as an Internet link and provided to trained experts for filling by high-risk groups.

In the next step, a qualitative study was designed and conducted to collect these groups’ opinions about the different stages of test distribution, how to do it, how to report its results, and how to connect these people to care and treatment centers. In this study, the purposive and voluntary sampling method was used so that seven people were selected from each of the studied high-risk groups, and then an interview was simultaneously conducted with these people using the focused group discussion (FGD) method. In order to collect information in this step, the fourth questionnaire was used to ask the high-risk groups for their opinions about how to distribute the test, how to perform it, how to report its results, and finally, its challenges.

Quality assurance is a set of activities before the study to collect the data with the highest possible quality. Also, all actions performed while collecting information or identifying and modifying any deviation from the executable guidelines are known as quality control. In this study, the following measures were used to ensure and control the quality of collected data: A standard executive protocol was developed, the authoritative administrative guide was evaluated using a pilot study, and an organizational observer was hired to monitor and assess the study process closely. Regular weekly meetings were held to review and resolve issues and repeatedly cleanse the data.

### Statistical analysis

For the quantitative part of the study, the data obtained from the questionnaires and checklists related to the level of acceptance and satisfaction were inserted into Excel. To compare the level of knowledge, satisfaction, and acceptance, chi-square test was used. Also, the comparison of the average feasibility between the high-risk groups was made using analysis of variance (ANOVA) and independent *t* test. These analyses were performed using STATA version 16.

MAXQDA version 10 software was also used to analyze the qualitative part. The speeches of the high-risk groups in the FGD interview were recorded and then transcribed into a Word file and finally coded and analyzed by the software.

## Results

A total of 930 eligible respondents were enrolled in the study, of whom 456 (49%) were female, 49 (5.3%) were transgender (98% of TGs were male to female), 804 (86%) were single, 568 (61%) were unemployed, 675 (73%) were diploma and above, 629 (67.6%) were living with a first-degree relative, and 174 (18.7%) were living alone. Their mean age was 33.63 years (10.54 SD) (the interquartile range [IQR] 18–77), and 406 people (62.10%) were less than 35 years old (Table 1).

**Table 1:** Sociodemographic characteristics of respondents of high-risk groups at baseline

Variables	Total	Iranian high-risk populations					
		FSWs (Number, %)		MSM (Number, %)		TG (Number, %)	
	Number (%)	FSWs 454 (48.8%)	Sexual partner 60 (6.5%)	MSM 352 (37.8%)	Sexual partner 16 (1.7%)	TGs 44 (4.7%)	Sexual partner 4 (0.4%)
*Location*							
Alborz	208 (22.4)	114 (25.1)	14 (23.3)	69 (19.6)	2 (12.5)	8 (18.2)	1 (25.0)
Tehran	722 (77.6)	340 (74.9)	46 (76.7)	283 (80.4)	14 (87.5)	36 (81.8)	3 (75.0)
Age (± SD)	33.63 (10.54)	39.19 (10.15)	35.22 (12.02)	27.79 (6.54)	27.00 (7.43)	23.59 (4.16)	30.75 (3.78)
*Age*							
18–25	238 (25.6)	39 (8.6)	15 (25.0)	144 (40.9)	9 (56.3)	31 (70.5)	0 (0.0)
26–35	339 (36.5)	131 (28.9)	16 (26.7)	169 (48.0)	6 (37.5)	13 (29.5)	4 (100.0)
36–45	215 (23.1)	164 (36.1)	18 (30.0)	33 (9.4)	0 (0.0)	0 (0.0)	0 (0.0)
46+	138 (14.8)	120 (26.4)	11 (18.3)	6 (1.7)	1 (6.3)	0 (0.0)	0 (0.0)
*Gender*							
Female	456 (49.0)	454 (100.0)	2 (3.3)	0 (0.0)	1 (6.3)	0 (0.0)	0 (0.0)
Male	425 (45.7)	0 (0.0)	58 (96.7)	349 (99.1)	14 (87.5)	0 (0.0)	3 (75.0)
Transgender	49 (5.3)	0 (0.0)	0 (0.0)	3 (0.9)	1 (6.3)	44 (89.8)	1 (25.0)
*Sex reassignment therapy*							
Male to female	1 (2.0)	0 (0.0)	0 (0.0)	0 (0.0)	1 (100.0)	0 (0.0)	0 (0.0)
Female to male	48 (98.0)	0 (0.0)	0 (0.0)	3 (100.0)	0 (0.0)	44 (100.0)	1 (100.0)
*Marital status*							
Married	126 (13.5)	90 (19.8)	19 (31.7)	14 (4.0)	1 (6.3)	0 (0.0)	2 (50.00)
Single	804 (86.5)	364 (80.2)	41 (68.3)	338 (96.0)	15 (93.8)	44 (100.0)	2 (50.00)
*Occupation*							
Unemployed	568 (61.1)	401 (88.3)	15 (25.0)	116 (33.0)	3 (18.8)	33 (75.0)	0 (0.0)
Employed	362 (38.9)	53 (11.7)	45 (75.0)	236 (67.0)	13 (91.3)	11 (25.0)	4 (100.0)
*Income*							
Less than 1 M*	14 (4.0)	4 (8.0)	2 (0.04)	7 (0.03)	0 (0.0)	1 (0.09)	0 (0.0)
1–2 M	65 (18.0)	16 (30.0)	2 (0.04)	43 (0.18)	1 (0.08)	3 (0.28)	0 (0.0)
2–4 M	147 (41.0)	30 (57.0)	18 (0.4)	88 (0.37)	8 (0.62)	3 (0.28)	0 (0.0)
More than 4 M	136 (38.0)	3 (6.0)	23 (0.51)	98 (0.42)	4 (0.31)	4 (0.37)	4 (100.0)
*Education*							
Under Diploma	255 (0.27)	202 (0.44)	22 (0.37)	21 (6.0)	1 (6.0)	9 (20.0)	0 (0.0)
Diploma and above	675 (0.73)	252 (0.56)	38 (0.63)	331 (94.0)	15 (94.0)	35 (80.0)	4 (100.0)
*Residence status*							
Homeless	18 (1.9)	16 (3.5)	0 (0.0)	2 (6.0)	0 (0.0)	0 (0.0)	0 (0.0)
Living with a partner	57 (6.1)	26 (5.7)	4 (6.7)	18 (5.1)	2 (12.5)	7 (15.9)	0 (0.0)
Living with a second-degree relative	16 (1.7)	3 (0.7)	5 (8.3)	8 (2.3)	0 (0.0)	0 (0.0)	0 (0.0)
Living with a first-degree relative	629 (67.6)	315 (69.4)	49 (81.7)	236 (67.0)	10 (62.5)	16 (36.4)	3 (75.0)
Living with friends	33 (3.5)	4 (0.9)	0 (0.0)	19 (5.4)	1 (6.3)	8 (18.2)	1 (25.0)
Living alone	174 (18.7)	88 (19.4)	2 (3.3)	68 (19.3)	3 (18.8)	13 (29.5)	0 (0.0)
Other	3 (0.3)	2 (0.4)	0 (0.0)	1 (0.3)	0 (0.0)	0 (0.0)	0 (0.0)

FSWs, Female Sex Workers; MSM, Men who have sex with Men; TGs, Transgenders

*Million Rials

Thirty-two percent of respondents had ever heard of HIV testing, of whom 61% and slightly more than 20% had received information from friends and counseling centers of behavioral diseases, respectively. 4% of participants had an HIV self-test in the past. When asked about what kind of support they would need to perform the HIV self-test, approximately 50% and 23.4% of participants indicated a need for the test educational counseling and confirmatory tests, respectively (Table 2).

**Table 2:** Knowledge levels of respondents of high-risk groups about HIV self-tests at baseline

Variables	Total	Iranian high-risk populations						*P* value*
		FSWs		MSM		TGs		
	Number (%)	FSWs (%)	Sexual partner (%)	MSM (%)	Sexual partner (%)	TGs (%)	Sexual partner (%)	
*Having knowledge about self-tests*								0.030
Yes	298 (32.0)	134 (29.5)	16 (26.7)	124 (35.2)	9 (56.3)	12 (27.3)	3 (75.0)	
No	632 (68.0)	320 (70.5)	44 (73.3)	228 (64.8)	7 (43.8)	32 (72.7)	1 (25.0)	
*How did you get information on the HIV self-test?*								0.792
Internet	11 (0.04)	0 (0.0)	0 (0.0)	10 (0.08)	0 (0.0)	1 (0.8)	0 (0.0)	
Family	11 (0.04)	4 (0.03)	6 (0.38)	1 (0.01)	0 (0.0)	0 (0.0)	0 (0.0)	
Friends	186 (0.61)	128 (0.9)	7 (0.44)	44 (0.35)	1 (0.13)	6 (0.50)	0 (0.0)	
Social networks	18 (0.06)	1 (0.01)	0 (0.0)	15 (0.12)	1 (0.13)	1 (0.08)	0 (0.0)	
Counseling Centers of Behavioral Diseases	79 (0.26)	7 (0.05)	3 (0.18)	56 (0.44)	6 (0.75)	4 (0.33)	3 (100.0)	
*Have you ever had an HIV self-test?*								0.550
Yes	38 (4.1)	17 (3.7)	2 (3.3)	16 (4.5)	2 (12.5)	1 (2.3)	0 (0.0)	
No	889 (95.6)	437 (96.3)	58 (96.7)	334 (94.9)	14 (87.5)	42 (95.5)	4 (100.0)	
*Which services would you like to receive after your HIV test?*								0.691
Confirmatory tests	218 (23.4)	134 (129.5)	21 (35.0)	58 (16.5)	0 (0.0)	5 (11.4)	0 (0.0)	
Educational advice	471 (50.6)	254 (55.9)	31 (51.7)	163 (46.3)	5 (31.3)	14 (31.8)	4 (100.0)	
Psychiatric counseling	139 (14.9)	33 (73)	4 (6.7)	78 (22.2)	5 (31.3)	19 (43.2)	0 (0.0)	
Other	102 (11.0)	33 (7.3)	4 (6.7)	53 (15.1)	6 (37.5)	6 (13.6)	0 (0.0)	

FSWs, female sex workers; MSM, men who have sex with men; TGs, transgenders

*Chi-square test

About 80% of participants did their test between 6 am and 6 pm. Virtually 79% of participants reported their HIV self-test results in the first 24 h. Almost 68% of the participants reported their test results by visiting counselors at the HIV/AIDS self-test centers. A total of 65% of the participants stated that they used educational aids to perform the test, of whom 37% preferred to use face-to-face counseling when receiving an HIV self-test and 16% preferred to use a pamphlet. The average satisfaction score with teaching aids was high (6.66 ± 0.65 SD). Although only 2% of participants used online consulting with experts, the average satisfaction score with online consultants was very high (6.86 ± 0.36 SD) (Table 3).

**Table 3:** Use of educational resources and the report of HIV self-test results by high-risk groups

Variables	Total	Iranian high-risk population						*P* value*
		FSWs		MSM		TGs		
	Number (%)	FSWs (%)	Sexual partner (%)	MSM (%)	Sexual partner (%)	TGs (%)	Sexual partner (%)	
*When did you take the test?*								0.887
6 am to 12 pm	380 (40.9)	205 (45.2)	35 (58.3)	114 (32.4)	9 (56.3)	16 (36.4)	1 (0.25)	
12 pm to 6 pm	351 (37.7)	217 (47.8)	9 (15.0)	108 (30.7)	3 (18.8)	12 (27.3)	2 (0.50)	
6 pm to 12 pm	165 (17.7)	28 (6.2)	15 (25.0)	106 (30.1)	3 (18.8)	13 (29.5)	0 (0.0)	
12 pm to 6 am	7 (0.08)	2 (0.4)	1 (1.7)	4 (1.1)	0 (0.0)	0 (0.0)	0 (0.0)	
*When did you report the test result?*								0.332
= < 24 h	689 (78.9)	289 (63.9)	51 (85.0)	294 (96.4)	15 (100.0)	37 (97.4)	3 (100.0)	
24–48 h	114 (13.1)	104 (23.0)	6 (10.0)	4 (1.3)	0 (0.0)	0 (0.0)	0 (0.0)	
> 48 h	70 (8.0)	59 (13.1)	3 (5.0)	7 (2.3)	0 (0.0)	1 (2.6)	0 (0.0)	
*How did you report the test result?*								0.401
The questionnaire link	1 (0.00)	0 (0.0)	0 (0.0)	0 (0.0)	1 (6.0)	0 (0.0)	0 (0.0)	
The online consultant	9 (0.00)	1 (0.0)	0 (0.0)	7 (2.0)	1 (6.0)	0 (0.0)	0 (0.0)	
Calling the counselor at the HIV/AIDS self-test center	252 (0.28)	140 (30.9)	24 (40.0)	77 (23.0)	1 (6.0)	10 (24.4)	0 (0.0)	
Visiting the counselor at the HIV/AIDS self-test center	610 (0.68)	311 (68.8)	36 (60.0)	220 (66.0)	12 (80.0)	28 (68.3)	3 (100.0)	
Being followed by the study counselors (inactive follow-up)	32 (0.04)	0 (0.0)	0 (0.0)	29 (8.7)	0 (0.0)	3 (7.0)	0 (0.0)	
*Did you refer to educational aids (the site, pamphlet, etc.) to complete your information?*								0.444
Yes	607 (67.0)	343 (75.9)	36 (60.0)	196 (57.8)	9 (60.0)	22 (54.0)	1 (33.3)	
No	303 (33.0)	109 (24.11)	24 (40.0)	143 (42.2)	6 (40.0)	19 (46.3)	2 (66.7)	
*Which teaching aid do you prefer to use?*								0.088
Pamphlet	149 (25.0)	59 (17.2)	7 (19.4)	72 (37.0)	1 (11.0)	10 (46.0)	0 (0.0)	
In-person counseling when receiving a test	346 (57.0)	241 (70.2)	18 (50.0)	73 (37.0)	6 (67.0)	7 (32.0)	1 (100.0)	
Web site and educational video	98 (16.0)	41 (11.9)	10 (28.0)	41 (21.0)	2 (22.0)	4 (18.0)	0 (0.0)	
Other	14 (2.0)	2 (0.0)	1 (3.0)	10 (5.0)	0 (0.0)	1 (5.0)	0 (0.0)	
*Did you call the introduced online expert for more information or ask a question?*								0.330
Yes	14 (2.0)	0 (0.0)	1 (1.7)	11 (3.0)	1 (6.6)	1 (2.4)	0 (0.0)	
No	896 (98.0)	452 (100.0)	59 (98.3)	328 (96.8)	14 (93.3)	40 (97.6)	3 (100.0)	
*Were you satisfied with the introduced online expert?*	6.86 (0.36)	–	6.0 (0.0)	7.0 (0.0)	7.0 (0.0)	6.0 (0.0)	–	0.901
*Were you satisfied with the teaching aids?*	6.66 (0.65)	6.73 (0.57)	6.75 (0.50)	6.57 (0.72)	6.22 (1.39)	6.41 (0.73)	6.00 (0.0)	0.882

FSWs, female sex workers; MSM, men who have sex with men; TGs, transgenders

*Chi-square test

The approval of the use of HIV self-tests in Iranian high-risk groups was significantly high so that 99% of participants answered yes in response to the question “in general, was it acceptable for you to perform the test in this way?” and 98% of them said they would like to retake this test. Ninety-one percent of the participants also stated they would recommend this test to their friends and sexual partners. Sixty-seven percent of participants preferred to take the test with a reliable consultant, and 60% preferred taking the test alone. In response to the question “where do you want to get the test?”, 51%, 48%, 45%, and 37% of the participants chose drug stores, counseling centers for behavioral diseases, comprehensive health service centers, and online stores, respectively (Table 4).

**Table 4:** Results of the acceptability of using HIV self-tests in Iranian high-risk groups

Variables	Total	Iranian high-risk population						*P* value*
		FSWs		MSM		TGs		
	Number (%)	FSWs (%)	Sexual partner (%)	MSM (%)	Sexual partner (%)	TGs (%)	Sexual partner (%)	
*In general, was it acceptable for you to perform the test in this way?*								0.880
Yes	903 (99.2)	450 (99.9)	60 (100.0)	335 (98.8)	15 (100.0)	40 (98.0)	3 (100.0)	
No	7 (0.8)	2 (1.0)	0 (0.0)	4 (1.00)	0 (0.0)	1 (2.00)	0 (0.0)	
*Would you like to use this test again?*								0.794
Yes	881 (98.4)	447 (99.1)	6 (100.0)	324 (98.8)	14 (93.3)	33 (86.8)	3 (100.0)	
No	14 (1.5)	4 (0.9)	0 (0.0)	4 (1.2)	1 (6.7)	5 (13.1)	0 (0.0)	
In whose presence do you prefer to take the test? *								0.099
Alone	550 (60.0)	297 (66.0)	34 (57.0)	189 (56.0)	10 (67.0)	18 (44.0)	2 (50.0)	
A family member	75 (8.0)	65 (14.0)	1 (2.0)	7 (2.0)	0 (0.0)	2 (5.0)	0 (0.0)	
A friend	162 (18.0)	72 (16.0)	5 (2.0)	66 (19.0)	4 (27.0)	15 (37.0)	0 (0.0)	
A reliable consultant	607 (67.0)	317 (70.0)	37 (2.6)	218 (64.0)	11 (73.0)	22 (54.0)	2 (50.0)	
Others	4 (0.0)	0 (00.0)	0 (2.0)	3 (1.0)	0 (0.0)	1 (2.0)	0 (0.0)	
Where do you want to get the self-test?*								0.551
Drug store	464 (51.0)	241 (53.0)	31 (52.0)	169 (50.0)	6 (40.0)	15 (37.0)	2 (67.0)	
Online store	337 (37.0)	177 (39.0)	14 (23.0)	127 (37.0)	6 (40.0)	13 (32.0)	0 (0.0)	
Counseling Centers of Behavioral Diseases	441 (48.0)	138 (31.0)	18 (30.0)	247 (73.0)	12 (80.0)	23 (56.0)	3 (100.0)	
Comprehensive health service centers	409 (45.0)	321 (71.0)	22 (37.0)	60 (18.0)	2 (13.0)	4 (10.0)	0 (0.0)	
Hangouts	115 (13.0)	41 (9.0)	7 (12.0)	58 (17.0)	0 (0.0)	8 (20.0)	1 (33.0)	
Peers	124 (14.0)	8 (2.0)	2 (3.0)	100 (29.0)	2 (13.0)	12 (29.0)	0 (0.0)	
Others	19 (2.0)	14 (3.0)	1 (2.0)	4 (1.0)	0 (0.0)	0 (00.0)	0 (0.0)	
*Do you recommend this test to your other friends or partners?*								0.550
Yes	828 (91.0)	399 (88.0)	53 (88.0)	323 (95.0)	14 (93.0)	36 (88.0)	3 (100.0)	
No	25 (3.0)	10 (2.0)	1 (2.0)	11 (4.0)	0 (0.0)	3 (7.0)	0 (0.0)	
I don’t know	57 (6.0)	43 (10.0)	6 (10.0)	5 (1.0)	1 (7.0)	2 (5.0)	0 (0.0)	

FSWs, female sex workers; MSM, men who have sex with men; TGs, transgenders

*Selecting multiple choices is leading to the result of more than 100%, *Chi-square test

### Reactive participants and connecting them to the counseling centers

Overall, 14 participants (1.5%) had reactive outcomes, of whom 10, 3, and 1 person were in the high-risk groups of MSM, TGs, and FSWs, respectively. They referred to the counseling centers for behavioral diseases for confirmatory tests. The two participants’ confirmatory test was negative. Individuals were introduced to the health center with a confirmed test to receive services (Table 5).

**Table 5:** Reactive participants and connecting them to counseling centers

Variables	Number (%)
*What was your HIV self-test result?*	
Negative	888 (95.4)
Positive (Reactive)	14 (1.5)
No answer no response	28 (3.1)
*Would you like to go to a trusted counseling center for a confirmatory test? (For positive participants)*	
Yes	12 (86)
No/No answer	2 (14)
*Would you like to talk to a trusted expert before having a confirmation test? (For positive participants)*	
Yes	10 (71)
No answer/No response	4 (29)

### Feasibility of the use of HIV self-tests in high-risk groups

The feasibility of using HIV self-tests in Iranian high-risk groups was significantly high. Ninety-seven percent of participants did not have any confidentiality problems while preparing or performing the test. In general, feasibility was assessed based on five questions. The overall feasibility score was 6.33 (0.824 SD). Totally, in response to the question “how accurate is the test result?” participants’ average score was 6.10 out of 7 points. Taking tests, reading HIV test results, finding a safe place to do the test, and accessing HIV self-testing showed a high average (Table 6).

**Table 6:** Results of the feasibility study of using HIV self-test by high-risk groups

	Iranian high-risk population						*P* value**
	FSWs		MSM		TGs		
	FSWs [Mean (SD)]	Sexual partners [Mean (SD)]	MSM [Mean (SD)]	Sexual partners [Mean (SD)]	TGs [Mean (SD)]	Sexual partners [Mean (SD)]	
How accurate is the test result?	6.17 (1.058)	6.13 (1.016)	6.00 (1.179)	5.73 (1.387)	6.10 (0.800)	6.33 (0.577)	0.339
*P* value*	0.002		0.033		0.021		
How easy was it for you to access your HIV self-test?	5/70 (1/872)	5/28 (2.293)	6/28 (1/141)	5/67 (1.496)	6/32 (1/192)	5/67 (1/528)	0.221
*P* value*	0.044		0.003		0.002		
How easy was it for you to do the HIV self-test yourself?	6/42 (1/134)	6/37 (1.089)	6/74 (0/573)	6/73 (0.594)	6/78 (0/571)	7/00 (0/000)	0.033
*P* value*	0.042		0.002		0.001		
How easy was it for you to read the HIV self-test results?	6/44 (1/136)	6/35 (1.132)	6/74 (0/605)	6/87 (0.352)	6/61 (0/666)	7/00 (0/000)	0.002
*P* value*	0.003		0.001		0.044		
How easy was it for you to find a safe place to test?	6/59 (0/855)	6/10 (1.285)	6.50 (0/905)	6/47 (0.743)	6/56 (0/867)	6/67 (0/577)	0.301
*P* value*	0.320		0.030		0.043		
Total	6.26 (0.960)	6.05 (1.001)	6.45 (0.569)	6.29 (0.692)	6.47 (0.588)	6.53 (0.503)	0.049
*P* value*	0.002		0.001		0.001		

FSWs, female sex workers; MSM, men who have sex with men; TGs, transgenders

*Independent *T* test

**ANOVA test

### Qualitative results

Six to 8 high-risk groups (MSM, FSWs, and TGs) were separately selected, and interviews were conducted with each population as a focused group discussion (FGD). All results were analyzed using content analysis (Table 7). After interviewing and analyzing the findings, the main challenges in distributing and accessing the HIVST, consulting on how to perform the HIVST, announcing the HIVST result, and connecting to healthcare centers were summarized (Table 7). Proposed solutions to address these challenges by high-risk groups (MSM, FSWs, and TGs) are presented in Table 7.

**Table 7:** Result of qualitative study analysis (challenges of HIV self-testing and proposed solutions)

Challenges	Proposed solutions
Distribution and access to HIVST	Provision of the HIVST in selected centers (currently, this method is suitable):
	Selecting centers with appropriate space and maintaining confidentiality with the help of trained experts and peers
	Access to these centers should be as easy as possible
	Provision of the HIVST in hangouts:
	Distribution of the HIVST by peers in hangouts and high-risk population gathering places
	Establishing the HIVST provision centers in places close to hangouts
	Provision of the HIVST in pharmacies
	Provision of the HIVST in online stores
	Elimination of legal barriers to distribution or access to HIVST
Counseling on how to perform the HIVST	In-person or face-to-face consultation:
	Setting up or developing centers for the distribution of HIVST with the presence of trained experts or using trained peers to provide sufficient and complete explanations about HIVST, how to do the test, and receive results
	More emphasis on how to perform the HIVST and how to read the results
	Online counseling
	Launching training webinars and online training Web sites with experts or trained peers
	Preparation and distribution of pamphlets and educational brochures during HIVST distribution or online pamphlets and brochures for high-risk groups
How to do the HIVST	Providing appropriate and sufficient training on how to perform the HIVST
	More appropriate distribution of educational materials related to how to perform HIVST
Announcing the HIVST result	Receiving test results in person or online if a suitable platform is prepared
	Setting up the monitoring system for high-risk groups after receiving HIVST
	Setting up a system of psychological and psychiatric counseling for high-risk groups (online or in-person) after receiving the HIVST results
Connection with treatment care centers	Providing appropriate training to gain the trust of high-risk groups to refer healthcare centers
	Providing incentives and gifts for referring to healthcare centers
	Setting up healthcare centers in the closest and most accessible places for high-risk groups
	Providing free or low-cost medical services in healthcare centers

## Discussion

This study aimed to evaluate the feasibility of using rapid HIV self-tests in high-risk populations. The results showed high acceptance of HIV self-tests (HIVST) in high-risk groups such as MSM, FSWs, and TGs in Iran, and on the other hand, most of the high-risk groups participating in the present study, such as TGs, MSM, and FSWs, confirmed the simplicity of performing the test. They also confirmed how the test was presented (the facility-based method). The present study results align with previous studies [24, 27, 29, 31–33].

More than 20 countries worldwide have accepted the policies concerning HIVST announced by the WHO. They have taken the necessary measures to implement this test in their own country. HIVST is currently presented in different ways in the world. The first method is to go to clinics to do the test (clinic-based testing or CBT) or provide the test from private or public pharmacies (the facility-based method). In this method, whole groups receive the test in the center or at their home by referring to selected centers and receiving face-to-face consultations, pamphlets, and training brochures to perform the test. The second way of presenting HIVST worldly is access to the tests in public places, such as buses, subways, train stations, public hospitals, or shops across the city, province, or country. This method is known as the community-based method. In this way, high-risk groups can receive and perform the test anytime and anywhere. The third method to present HIVST to high-risk groups is visiting reputable Web sites to purchase and order the test online. This method is known as online shopping [33–38].

According to the results of this study in Iran, high-risk groups have considered the facility-based method better for receiving HIVST. This result was confirmed by the results of previous studies, while most of them used the facility-based method to distribute and present HIVST [39–41]. The qualitative interview conducted in the present research showed that from the perspective of most high-risk groups, the best and easiest way to access HIVST in the country would be to provide tests in service delivery centers such as counseling centers for behavioral diseases and other related ones. Of course, other possible methods, such as offering tests in hangouts and pharmacies and providing the necessary facilities for online test sales, can also be helpful in Iran.

A notable result in the present study was the knowledge of high-risk groups and their partners on HIVST. The results showed that 68% of the high-risk groups in the study did not have sufficient knowledge about HIVST. The results were similar for the different vital groups participating in the study, and all high-risk groups and their partners had insufficient information and knowledge about HIVST. Based on these results, it is necessary to design and implement an accurate and easy plan to increase the knowledge of these groups and their partners in the country. These programs can be done with inter-sectorial cooperation and the cooperation of the high-risk groups. High-risk individuals and their partners can play an influential role in increasing other high-risk groups' awareness and knowledge about HIVST. According to this research, most high-risk groups participating in this study have received little information about HIVST from their friends. Also, to increase the awareness of critical groups, qualitative interviews showed that the whole groups were more focused on in-person and in absentia counseling. These people emphasized that centers for distributing self-tests should be set up with qualified and trained experts or the high-risk people themselves to provide complete training on self-tests. These groups also believed that holding online and in-person workshops and webinars about HIVST could significantly raise awareness. These methods can effectively increase the high-risk groups' awareness and knowledge of HIVST. The results of previous studies have also emphasized these methods to increase the awareness of critical people [39–45]. For example, Tucker JD et al. have noted that social marketing can be very effective in training people about receiving and performing HIVST and evaluating their results [32]. In addition, the advantages of this test, such as better preservation of the confidentiality and secrets of high-risk groups and the absence of blood or blood sampling for testing, can encourage high-risk groups to receive HIVST [34–37].

None of the specific policies and plans of countries have been developed and designed for the process after the test, and the taken actions are scattered and irregular. A limited number of countries, such as the USA or the European countries, have hired several trained experts who follow up on test results in high-risk groups using fixed or mobile phone numbers (the passive method) or in-person sessions (the active method) and ask these people to go to service centers to continue the process and receive medical services. This method was also used in the present study, and the entire groups were satisfied with this process. Therefore, it can be concluded that the facility-based method and actively and passively following the results are the most appropriate methods for presenting HIVST in Iran.

Based on the results of this study, it can be concluded that HIVST can be presented to the high-risk groups of MSM, FSWs, and TGs, or other high-risk groups such as PWID, prisoners, etc. With this test, we can even reach the high-risk groups who have not been before tested for HIV at all because most of the high-risk participants in the present study mentioned that they had never been tested for HIV before. In Iran, regular and specific services related to HIV are not currently provided to high-risk groups, especially to transgender people, MSM, or FSWs. So, the distribution of HIVST in these groups can be a valuable and practical step toward preventing or controlling HIV infection.

This research was the first study in Iran to evaluate the feasibility of HIVST in critical groups of MSM, TGs, and FSWs. The study also focused on the groups most at risk for HIV. One of the limitations of this study has been the low sample size of the transgender group, so it is necessary to conduct further studies on using HIVST with larger sample sizes of this group. In this study, through the Welfare Organization, non-governmental organizations, the Ministry of Health and Medical Education, and finally through the peers of the transgender groups, they were invited to receive HIVST. However, a small number of these people referred to receive them. A variety of reasons may be considered for this, such as the corona pandemic, the transgender groups' fear and anxiety regarding the infection detection by HIVST, the stigma or discrimination in selected centers when receiving HIVST, and the lack of transgender groups' trust in HIVST providers in selected centers.

Nevertheless, the results related to the TG group showed they were satisfied with HIVST. On the other hand, this study was a pilot to investigate the feasibility of using HIVST in the whole group. Therefore, further studies are needed to be separately done in these groups, and other studies with larger sample sizes can be designed and implemented in other high-risk groups such as PWIDs, prisoners, or even the general population.

## Conclusion

HIVST in the groups of FSWs and MSM had reasonable acceptability according to the present study results, and this test acceptance was good in these groups. However, for better access for these groups to HIVST, more appropriate and accurate planning is needed on how to provide and distribute the test, how to provide appropriate training related to the test, how to follow people's test results, and finally, how to refer and link these people to care and treatment centers. In Iran, these groups are less likely than other populations to go to service centers to do HIV self-tests and receive services because there are still stigma and discrimination related to HIV in Iran. The study of the distribution and feasibility of HIVST in high-risk groups or at-risk populations can help reach out to people who do not refer for testing because of stigma, discrimination, or fear of disclosure.

## Data Availability

The datasets generated and analyzed during the current study are not publicly available due to their sensitive and potentially personally identifiable nature. However, they are available from the corresponding author on reasonable requests.

## Abbreviations

FSW: Female sex workers
HIVST: HIV self-testing
IDUs: Injecting drug users
MSM: Men who have sex with men
SD: Standard deviation
TG: Transgender
UN: United Nations
